# A retrospective study of baseline peritoneal transport character and left ventricular hypertrophy in incident peritoneal dialysis patients: interrelationship and prognostic impacts

**DOI:** 10.1080/0886022X.2022.2148536

**Published:** 2023-01-16

**Authors:** Yi Wang, Guansen Huang, Xiaoyan Ma, Xiujuan Zang, Shoujun Bai, Yakun Wang, Lin Du, Zexin Lv, Jinqing Li, Hui Chen, Yan Hu, Yingfeng Shi, Xun Zhou, Min Tao, Shougang Zhuang, Na Liu

**Affiliations:** a Department of Nephrology, Shanghai East Hospital, Tongji University School of Medicine, Shanghai, China; b Department of Nephrology, Shanghai Songjiang District Central Hospital, Shanghai, China; c Department of Nephrology, Qingpu Branch of Zhongshan Hospital Affiliated to Fudan University, Shanghai, China; d Department of Medicine, Rhode Island Hospital and Alpert Medical School, Brown University, Providence, RI, USA

**Keywords:** Peritoneal dialysis, cardiovascular disease, left ventricular hypertrophy, retrospective study

## Abstract

**Background:**

Left ventricular hypertrophy is associated with adverse outcomes among peritoneal dialysis patients. The aim of this study was to evaluate the prognostic impact of baseline left ventricular hypertrophy and its relationship with baseline peritoneal transfer characteristics in peritoneal dialysis patients.

**Methods:**

We enrolled 151 incident peritoneal dialysis patients to perform a multicentric retrospective cohort study since January 1, 2017 to January 31, 2021. Patients were grouped based on baseline dialysate-to-plasma creatinine ratio at 4 h as follows: low (<0.50), low average (0.5–0.64), high average (0.65–0.80) and high (≥0.81). Echocardiography and clinic data were recorded yearly. The Cox proportional hazards models and competing risk model were used to evaluate patients’ survival. Generalized linear mixed models were performed to explore risk factors associated with left ventricular hypertrophy.

**Results:**

During a median follow-up period of 33 months (range, 16–48 months), 21 (13.9%) patients died, including 16 (10.60%) cardiovascular deaths. Controlling the competing risks of switching to hemodialysis, kidney transplantation and loss to follow-up, baseline left ventricular hypertrophy was an independent risk factor for all-cause mortality (subdistribution hazard ratio, 2.645; 95% confidence interval, 1.156–6.056; *p* = 0.021). Baseline high and high average transport status were positively related to left ventricular mass index and left atrium diameter 2 years after PD initiation.

**Conclusion:**

Baseline fast peritoneal solute transport rate may be an effect factor for aggravating left ventricular hypertrophy which predicted poor outcomes for peritoneal dialysis patients. The findings offered important ideas for further prospective intervention study.

## Introduction

Cardiovascular disease (CVD) is a common complication and has been considered as an important reason of death in patients with maintenance dialysis. Compared with hemodialysis (HD), peritoneal dialysis (PD) may have an advantage of survival in a relatively early period [1,2]. Nonetheless, approximately 1–2 years after PD initial, the patient’s survival often be comparable to or even poorer than HD [1,3]. A prospective cohort study demonstrated that patients with heart failure who initially received PD therapy had a lower survival rate than those who received HD [4]. These deleterious consequences could be explained partly by altered peritoneal transport patterns occurring over time.

Long-term success of PD is depended on the ability of the peritoneal membrane to provide adequate solute clearances and ultrafiltration over time. Peritoneal solute clearances is described as peritoneal solute transport rate (PSTR), which can be measured by the peritoneal equilibration test (PET) through dialysate-to-plasma (D/P) ratios of low-molecular-weight solutes [5]. Ultrafiltration failure is one of the consequences of fast solute transportation, which is the main limitation of long-term PD treatment. Fast PSTR accompanied by a rapid disappearing glucose osmosis gradient and increasing in protein loss, ultimately step in fluid overload and malnutrition [6]. Therefore, cardiovascular prognosis of patients with fast PSTR at baseline is more worthy of attention.

Left ventricular hypertrophy (LVH) is a major risk factor for cardiovascular complications and death in end stage kidney disease (ESKD) patients [7]. The prevalence of LVH is about 75% in patients with dialysis [8,9]. LVH and left atrium (LA) dilation now are risk factors closely tied to worse survival and cardiovascular outcomes [10,11]. Due to its convenience, echocardiography was a widespread way to evaluate cardiac structure and function noninvasively in PD patients. Adjusted with body surface area, left ventricular mass index (LVMI) can be an individualized way to assess left ventricular geometry and myocardial systolic and diastolic functions as well as their alternations in the follow-up visit. In previous studies, baseline fast peritoneal transport status was considered closely related to the increase of CVD occurrence in PD patients [12–14].

This present study focused on both baseline PSTR and echocardiography parameters, determining whether baseline fast PSTR and LVH could provide complementary predictive information on prognosis in PD patients in multi-center. Meanwhile, we detected the association between peritoneal transfer characters and LVH at the early stage of PD therapy.

## Materials and methods

### Study design and study population

The present study was a multicenter retrospective cohort study which recruited all the patients, aged between 18 and 75 years, who initiated continuous ambulatory peritoneal dialysis (CAPD) in four medical centers (Shanghai East Hospital Affiliated to Tongji University School of Medicine, Baoshan Branch of Shanghai First People’s Hospital, Shanghai Songjiang District Central Hospital and Qingpu Branch of Zhongshan Hospital Affiliated to Fudan University) since January 1^st^, 2017 to January 1^st^, 2019, and followed up until January 31^st^, 2021. All patients initiated CAPD with 1.5% or 2.5% dextrose PD fluid (Baxter Healthcare, Guangzhou, China), daily dialysis dose ranged from 6000 to 10000 mL. All the participants underwent a PET after the first month of PD treatment. Patients were excluded if they lacked complete echocardiography data, had previously received HD or kidney transplantation, had a history of malignant tumor, liver cirrhosis, active tuberculosis, acute myocardial infarction, severe heart valve lesions or peritonitis at the baseline. These enrolled patients were followed until cessation of PD, death, or on January 31st, 2021.

### Demographic and clinical data

Baseline demographic, medication history and clinical data were collected after the first month of PD treatment. Uncontrolled hypertension was defined as BP ≥140/90 mmHg. CVD included previous and present history of congestive heart failure, ischemic heart disease, or cerebrovascular disease. Baseline laboratory information at the first months after the initiation of CAPD, including hemoglobin (HB), serum albumin (ALB), high-sensitivity C-reactive protein (hs-CRP), corrected serum calcium (cSCa), phosphate (P), parathyroid hormone (PTH), uric acid (UA), total cholesterol (TC), triglyceride (TG), low-density lipoprotein (LDL) and high-density lipoprotein (HDL). The PD adequacy and residual renal function (RRF) were assessed by peritoneal and kidney Kt/V urea (urea clearance index) using data from 24-h dialysate and urine collections.

### Peritoneal equilibration test

According to Twardowski [5], a standard 4-h dwell period was used to perform PET test. Using 2.5% dextrose PD solution for a 2 L volume exchange, after 2.5% dextrose PD solution dwelling overnight (8–12 h). PD effluent sample was collected at 0, 2, 4 h of dwell and venous blood sample at 2 h, respectively. Both dialysate and plasma glucose and creatinine were measured. According to dialysate-to-plasma (D/P) creatinine ratio at 4 h (4 h D/P Cr), the patients were classified into four groups of peritoneal transport characteristics: low (L, < 0.50); low-average (LA, 0.50–0.64); high-average (HA, 0.65–0.80); high (H, > 0.81). The first PET test was defined as baseline data.

### Echocardiography

We collected echocardiography data of the first two consecutive years since PD initial. Comprehensive examination data of transthoracic two-dimensional echocardiography were collected by using Philips EpiQ-7 (Koninklijke Philips N.V., Netherlands) equipped with a multifrequency transducer (S5-1, 5 MHz). All images were obtained with standard techniques using M-mode, two-dimensional, and Doppler measurements in accordance with the American Society of Echocardiography guidelines (ASE). Echocardiographic measurements included left atrium diameter (LAD), left ventricular end-diastolic dimension (LVEDD), interventricular septum thickness (IVST) and posterior wall thickness (PWT). LV ejection fraction (LVEF) was calculated using the modified biplane Simpson’s method in apical two-chamber and four-chamber views. LVEF > 55% was defined as normal ventricle systolic function. Left ventricular mass (LVM) was calculated using the modification by Devereux et al. of the ASE cube formula: 0.8 × (1.04 × [LVEDd + IVST + PWT]^3^ − LVEDd^3^) + 0.6 [15]. LVMI was calculated according to the formula from linear dimensions and indexed to body surface area calculated using the formula: 0.0001 × 71.84 × (weight [kg])^0.25^ × (height [cm])^0.725^[16]. LVH was defined as LVMI > 131 g/m^2^ for men and > 100 g/m^2^ for women. LA enlargement（LAE） defined as LAD > 40 mm for men and LAD > 38 mm for women [17].

### Outcomes

The primary outcome was all-cause mortality, and the secondary one was cardiovascular mortality. For mortality analyses, patients were censored at the time of switching to HD treatment, kidney transplantation, lost to follow-up or the end of the study period (January 31st, 2021).

### Statistical analysis

Results were expressed as absolute number (percentage) for categorical variables, mean ± SD for continuous data and median (interquartile range) for nonnormally distributed continuous variables. Data distribution normality was evaluated by the Kolmogorov–Smirnov test. A comparison among the different peritoneal transport types was performed by one-way ANOVA (parametric distribution) or the Kruskal-Wallis test (non-parametric distribution), chi-square test was applied to compare categorical data. Linear regression analysis was used to definite the change slope of LVMI and LAD, the slope was expressed as the regression coefficient. Depending on whether the slope value is greater than 0, the changing trends of echocardiography parameters were differentiated as ‘rise’ or ‘fall’.

The Kaplan–Meier survival curves were drawn for each event of interest (overall death and cardiovascular death) and the log-rank test was used to compare curves. Univariate and multivariate Cox proportional hazards models were used to search significant risk factors associated with study outcomes, and group of baseline low average transporters was defined as reference. We used Z-score method to approximate normal data distribution. Variables that were statistically significant with a p-value < 0.2 in univariate Cox models were further assessed with multivariate Cox models using a forward stepwise method. The results were presented as adjusted hazard ratios (AHRs) with 95% confidence intervals (CIs). The Fine-Gray subdistribution hazards model was used to evaluate competing risks of censor events. The results were presented as subdistribution hazard ratios (SHRs) with 95% CIs. Generalized linear mixed models were used to explore risk factors associated with the elevated echocardiography parameters 2 years after PD initiation. All statistical analyses were performed by using SPSS software, version 23.0 (IBM SPSS, Chicago, IL, USA) and Stata/MP version17.0. A two-tailed *p*-value < 0.05 was considered statistically significant.

## Results

### Baseline characteristics of the entire cohort

**Figure 1.**
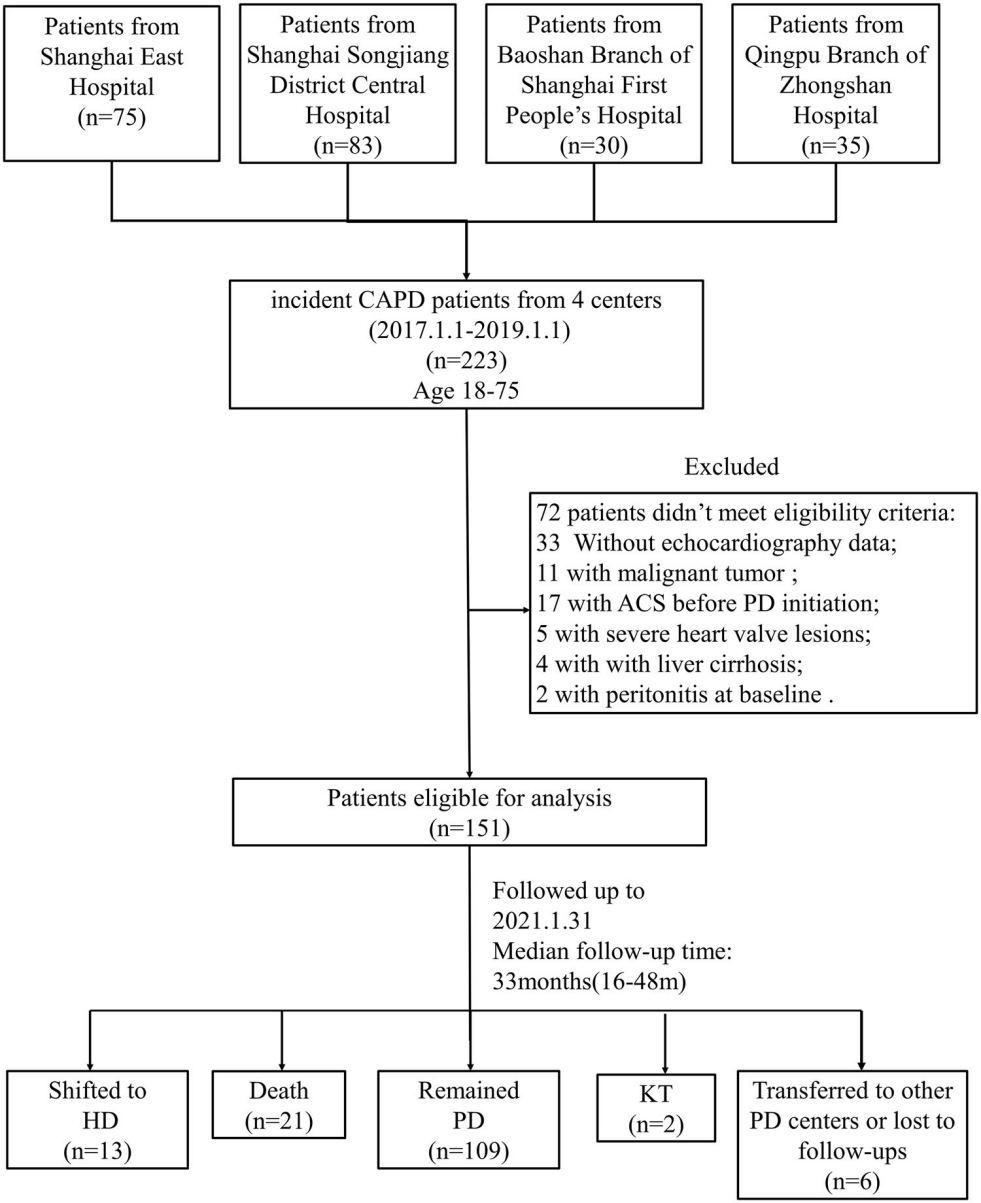
Flow chart of the participants in the study cohort. KT: kidney transplant; PD: peritoneal dialysis; HD: hemodialysis.

The flow chart of patients for the study was shown in Figure 1. 151 incident PD patients were enrolled into this retrospective study. The median follow-up time was 33 months (range, 16–48 months). The median age was 61 years (range, 52–68 years) old, and there were 82 males (54.30%). All patients were categorized by their baseline peritoneal transport status, 31 patients (20.53%) were classified as high peritoneal transporters (H group), 65 patients (43.05%) as high-average transporters (HA group), 46 patients (30.46%) as low-average transporters (LA group), and 9 patients (5.90%) as low transporters (L group). Compared with L and LA groups, less patients in group HA and H had their BP controlled (only 15.4% and 12.9%, respectively). Albumin levels in H group seemed to be significantly lower than in other groups while UA and cSCa levels was the opposite. The baseline characteristics of the participants were listed in Table 1.

**Table 1.** Baseline clinical parameters of 151 patients on continuous ambulatory peritoneal dialysis.

Parameters	Total population (*n* = 151)	L (*n* = 9)	LA (*n* = 46)	HA (*n* = 65)	H (*n* = 31)	*p* value
Age (y)	61.00 (52.00, 68.00)	61.00 (57.50, 67.50)	61.00 (52.00, 69.00)	60.00 (51.75, 66.00)	58.00 (48.00, 69.00)	0.438
Male n (%)	82 (54.3)	3 (33.3)	23 (50.00)	34 (52.3)	22 (71.0)	0.140
DM n (%)	58 (38.4)	6 (66.7)	21 (45.7)	18 (27.7)	13 (41.9)	0.061
RAAS Blockers n (%)	97 (64.2)	5 (55.6)	36 (78.3)	39 (60.00)	17 (54.8)	0.116
BP <140/90mmHg n (%)	30 (19.9)	2 (22.22)	14 (30.4)	10 (15.4)	4 (12.9)	0.170
BSA (m^2^)	1.69 ± 0.18	1.65 ± 0.11	1.67 ± 0.18	1.68 ± 0.20	1.73 ± 0.16	0.269
Weekly peritoneal Kt/V	1.56 (1.38, 1.71)	1.71 (1.60, 1.73)	1.55 (1.36, 1.69)	1.53 (1.34, 1.71)	1.55 (1.40, 1.67)	0.164
Weekly renal Kt/V	0.44 (0.08, 0.87)	0.48 (0.00, 0.74)	0.48 (0.22, 0.88)	0.48 (0.07, 0.90)	0.29 (0.00, 0.72)	0.299
4h D/P Cr	0.69 ± 0.13	0.47 ± 0.05*	0.59 ± 0.04	0.73 ± 0.04*	0.86 ± 0.03*	<0.01
Hb (mg/L)	99.71 ± 20.42	98.56 ± 26.50	101.18 ± 18.64	99.63 ± 19.53	98.08 ± 23.53	0.776
UA (μmol/L)	434.93 ± 100.91	475.74 ± 134.47	413.50 ± 87.27	426.48 ± 88.91	472.31 ± 122.21*	0.037
Alb (g/L)	33.06 (30.43, 35.12)	32.05 (30.43, 35.12)	34.58 (30.64, 37.00)	34.47 (31.00, 37.00)	32.00 (27.77, 34.35)*	0.033
hs-CRP (mg/L)	3.56 (1.46, 8.54)	5.00 (1.29, 12.78)	5.00 (1.60, 11.08)	2.53 (1.39, 5.00)	4.48 (1.46, 8.79)	0.351
TC (mmol/L)	4.20 (3.55, 4.93)	4.02 (3.10, 5.42)	4.19 (3.51, 5.50)	4.19 (3.80, 4.80)	4.32 (3.39, 5.28)	0.958
TG (mmol/L)	1.58 (1.12, 2.32)	2.21 (1.78, 2.63)*	1.66 (1.40, 2.51)	1.49 (1.03, 2.32)	1.35 (0.98, 2.00)	0.021
LDL (mmol/L)	2.77 (2.08, 3.58)	2.28 (1.70, 3.08)	2.52 (1.99, 3.42)	2.71 (2.21, 3.46)	2.53 (1.77, 3.91)	0.489
HDL (mmol/L)	1.01 (0.84, 1.16)	0.75 (0.68, 1.02)*	1.04 (0.90, 1.17)	1.05 (0.90, 1.26)	0.91 (0.76, 1.11)	0.004
PTH (ng/L)	199.60 (108.00, 324.00)	406.00 (249.00, 483.45)	165.50 (105.00, 304.00)	60.00 (142.35, 317.70)	58.00 (67.30, 330.05)	0.820
cSCa (mmol/L)	2.29 (2.194, 2.443)	2.35 (2.20, 2.56)	2.27 (2.17, 2.36)	2.28 (2.20, 2.41)	2.38 (2.24, 2.46)*	0.001
Serum P (mmol/L)	1.55 (1.28, 1.81)	1.41 (1.04, 1.70)	1.58 (1.37, 1.81)	1.56 (1.31, 1.93)	1.51 (1.27, 1.97)	0.907
HbAlc (%)	5.90 (5.20, 6.80)	6.30 (5.75, 6.58)	5.70 (5.00, 6.30)	5.90 (5.20, 6.90)	6.30 (5.45, 7.40)	0.074

*p < 0.05, compared with LA.

Abbreviations: L: low; LA: low average; H: high; HA: high average; DM: diabetes mellitus; RAAS blocker: renin-angiotensin-aldosterone system blocker; BP: blood pressure; BSA: body surface area; Kt/V: urea clearance index; 4 h D/P Cr: dialysate: plasma creatinine ratio at 4 h; Hb: hemoglobin; UA: uric acid; Alb, albumin; hs-CRP, high-sensitivity C-reactive protein; TC, total cholesterol; TG, triglyceride; LDL, low density lipoprotein; HDL, high density lipoprotein; PTH: parathyroid hormone; cSCa: corrected serum calcium; P: phosphate; HbA_1_c: glycosylated hemoglobin.

Note: cSCa = serum Ca + 0.8_x_ (4.0-serum ALB) [if serum ALB < 4g/dL].

### Echocardiographic characteristics of the entire cohort

Echocardiographic parameters at baseline and two-year follow-up were presented in Table 2. Regarding LA group as the reference, only baseline LAD was significantly higher in H group. While during the 2-year follow-up period, multiple echocardiographic parameters were found to be significantly altered in H group which presented with higher value of heart chamber size such as LVESV, LVEDD and LAD as well as progressive cardiac hypertrophy accompanied by deterioration of the left ventricular ejection fraction. Similarly, the same changes were also shown in HA group in 2^nd^ year. To show the changes more visual, variation of echocardiographic parameters about LVH was described as the slope of line regression based on the follow-up data. There were 94 (62.3%) and 80 (53.0%) patients had their LVMI and LAD elevated 2 years after PD initiation respectively. In comparison with LA group, the slopes of LVMI and LAD in H group indicated statistically differences as shown in Table 2.

**Table 2.** Baseline and follow-up visit clinical echocardiographic parameters of 151 patients on continuous ambulatory peritoneal dialysis.

Parameters	Total population (*n* = 151)	L (*n* = 9)	LA (*n* = 46)	HA (*n* = 65)	H (*n* = 31)
Baseline echocardiographic parameters					
LVEDD (mm)	50.00 (46.00, 53.00)	54.00 (51.50, 56.50)	50.00 (44.00, 53.00)	50.00 (46.00, 53.00)	50.00 (46.00, 53.00)
LVESD (mm)	31.00 (29.00, 34.00)	33.00 (32.25, 35.00)	31.00 (27.00, 33.00)	32.00 (30.00, 34.00)	31.00 (30.00, 34.00)
IVSD (mm)	9.00 (8.00, 10.00)	10.00 (8.50, 10.75)	9.00 (9.00, 10.00)	9.00 (8.00, 11.00)	9.00 (9.00, 10.00)
LVPWD (mm)	9.00 (8.00, 10.00)	10.00 (9.25, 10.75)	9.00 (8.00, 10.00)	9.00 (8.00, 10.00)	9.00 (8.00, 10.00)
LVEF (%)	65.00 (62.00, 70.00)	65.00 (60.50, 67.00)	66.00 (62.00, 72.00)	65.00 (61.00, 68.00)	66.00 (63.00, 70.00)
LVMI (g/m^2^)	102.99 (81.25, 121.71)	116.26 (103.89, 156.09)	99.47 (80.41, 119.92)	105.20 (85.16, 136.01)	93.85 (79.70, 119.90)
LAD (mm)	38.00 (36.00, 40.00)	39.00 (36.50, 41.00)	37.00 (35.00, 39.25)	38.00 (36.00, 41.00)	39.00 (38.00, 41.00)**
1-y echocardiographic parameters					
LVEDD (mm)	50.00 (46.00, 53.00)	52.50 (50.25, 56.50)	48.00 (44.75, 51.00)	50.00 (46.00, 52.00)	52.00 (48.00, 54.00)*
LVESD (mm)	32.00 (29.00, 35.00)	33.50 (30.75, 35.00)	30.00 (27.00, 33.00)	32.00 (30.00, 34.00)*	33.00 (30.00, 34.00)**
IVSD (mm)	10.00 (9.00, 11.00)	9.50 (9.00, 11.50)	10.00 (9.00, 11.00)	10.00 (9.00, 11.00)	10.00 (10.00, 12.00)
LVPWD (mm)	10.00 (9.00, 10.00)	9.50 (9.00, 10.00)	9.50 (8.00, 11.00)	10.00 (9.00, 10.00)	10.00 (9.00, 11.00)
LVEF (%)	64.00 (60.00, 68.00)	62.00 (54.50, 66.00)	63.00 (62.75, 69.25)	62.00 (60.00, 67.50)*	66.00 (58.00, 66.00)
LVMI (g/m^2^)	105.46 (92.43, 127.79)	123.86 (109.02, 142.71)	100.71 (78.47, 115.23)	105.47 (94.80, 131.30)	110.59 (93.28, 166.68)
LAD (mm)	39.00 (35.00, 42.00)	40.50 (37.75, 42.00)	40.50 (35.00, 40.25)	41.00 (35.00, 42.00)	40.00 (38.00, 44.00)**
2-y echocardiographic parameters					
LVEDD (mm)	51.00 (46.00, 54.00)	51.50 (50.25, 54.75)	49.00 (43.00, 52.00)	50.00 (47.00, 54.00)	54.00 (51.00, 57.00)**
LVESD (mm)	33.00 (29.00, 36.00)	35.00 (35.00, 35.00)	30.00 (28.00, 34.25)	33.00 (30.00, 36.50)	35.00 (33.00, 38.00)**
IVSD (mm)	10.00 (9.00, 11.00)	10.50 (9.00, 11.00)	9.00 (9.00, 11.00)	10.00 (9.00, 11.00)	11.00 (10.00, 12.00)*
LVPWD (mm)	10.00 (9.00, 11.00)	10.00 (9.00, 11.00)	9.00 (8.00, 10.00)	10.00 (9.00, 11.00)	10.00 (9.00, 12.00)*
LVEF (%)	63.00 (58.00, 67.00)	61.00 (50.00, 67.50)	66.00 (62.00, 70.00)	63.00 (59.50, 66.50)*	62.00 (56.00, 66.00)*
LVMI (g/m^2^)	114.51 (95.14, 143.38)	131.66 (107.49, 149.44)*	99.12 (78.33, 115.37)	115.28 (100.95, 139.89)**	151.93 (107.21, 186.75)**
LAD (mm)	40.00 (36.00, 42.00)	40.50 (39.25, 41.75)	38.00 (33.75, 41.25)	40.00 (36.00, 43.00)	41.00 (39.00, 44.00)**
Slope of 2y changes in echocardiographic parameters					
LAD slope (mm/y)	1.00 (0.00, 1.50)	0.75 (0.00,1.50)	0.50 (−0.50, 1.13)	0.50 (−0.50, 1.75)	1.50 (1.00, 2.00)**
LVMI slope (g/m^2^/y)	3.88 (−4.08, 21.14)	2.25 (−9.14, 16.59)	−1.77 −10.42, 6.31)	4.38 (−3.69, 17.35)	22.69 (15.35, 37.47)**
LVEF slope (%/y)	−1.00 (−3.13, 1.00)	−2.50 (−5.37, 2.00)	−0.50 (−2.63, 1.63)	−1.00 (−2.50, 1.00)	−2.00 (−4.00, 1.50)

*p < 0.05, compared with LA; **p < 0.01, compared with LA.

Abbreviations: LVEF: left ventricular ejection fraction; LAD: left atrial dimension; LVEDD: left ventricular end-diastolic dimension; LVH: left ventricular hypertrophy; LVMI: left ventricular mass index; IVSD: interventricular septal thickness at diastole; LVPWD: left ventricular posterior wall thickness; LVESD: left ventricular end-systolic diameter.

### Patient survival and cardiovascular survival

**Figure 2.**
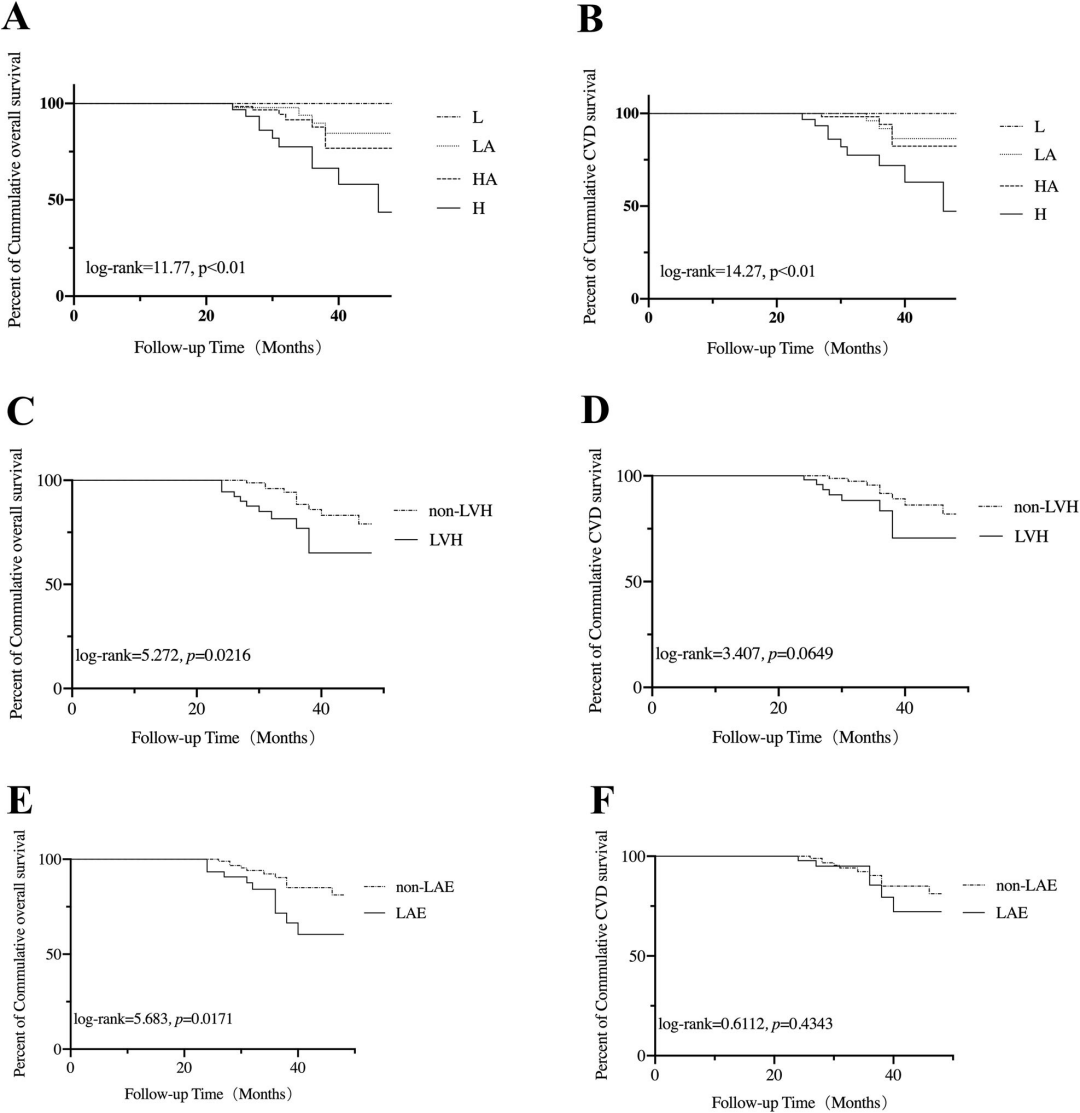
Kaplan-Meier analysis of overall survival and cardiovascular survival of the participants. (A) The overall survival of participants classified according to the type of peritoneal transportation. (B) The cardiovascular survival of the participants classified according to the type of peritoneal transportation. (C) The overall survival of the participants classified according to the diagnostic criteria for LVH by echocardiography. (D) The cardiovascular survival of the participants classified according to the diagnostic criteria for LVH by echocardiography. (E) The overall survival of the participants classified according to the diagnostic criteria for LAE by echocardiography. (F) The cardiovascular survival of the participants classified according to the diagnostic criteria for LAE by echocardiography. L: low; LA: low-average; HA: high-average; H: high; LVH: left ventricular hypertrophy; LAE: left atrial enlargement.

In this retrospective study, all-cause death in 21 (13.90%) and cardiovascular death in 16 (10.60%) subjects were observed. Details on all-cause and cardiovascular deaths were listed in Supplementary Table 1. Cumulative overall survival at 1, 2, 3 and 4 year was 100%, 98.0%, 84.5% and 74.6%, respectively, cumulative cardiovascular survival was 100%, 99.3%, 88.9% and 78.5%, respectively. Compared with the other groups, patient overall survival was significantly decreased in H group (*p* < 0.01) (Figure 2(A)). A higher cardiovascular mortality was also observed in H group (*p* < 0.01) (Figure 2(B)). Based on baseline LVMI, patients were divided into LVH and non-LVH group. It was noticed that patients in LVH group had higher all-cause mortality, but no significant statistical difference in CV survival. (*p =* 0.022 and *p =* 0.065, respectively) (Figure 2(C,D)). Similarly, patients were divided into LAE and non-LAE group based on baseline LAD. A significant decreased overall survival was observed in LAE group (*p =* 0.017) (Figure 2(E)), while CV survival did not differ between the two groups. (*p =* 0.434) (Figure 2(F)).

We performed univariate and multivariate Cox proportional hazard regression analysis to assess the risk factors for all-cause mortality. In the univariate model, baseline H transport status (*p =* 0.015) and hemoglobin (*p =* 0.036) was associated with all-cause mortality. When LVMI and LAD was converted to a binary classification variable according to the diagnostic criteria of LVH and LAE by echocardiography, we found LVH (*p =* 0.028) and LAE (*p =* 0.023) were risk factors for all-cause mortality. Similarly, the univariate Cox regression analysis also showed positive relationship between baseline H transport status and cardiovascular mortality (*p =* 0.014). Then, a multivariate Cox model was constructed. Baseline LVH predicted an increased risk of all-cause death (AHR, 3.716; 95% CI, 1.432–9.640; *p =* 0.007) and cardiovascular death (AHR, 3.923; 95% CI, 1.224–12.567; *p =* 0.021). Meanwhile, hemoglobin appeared predicting a decreased risk of all-cause death (AHR, 0.971; 95% CI, 0.946–0.997; *p =* 0.026) and cardiovascular death (AHR, 0.962; 95% CI, 0.934–0.992; *p =* 0.013). Baseline H transport status and LAE were no longer significant for outcome events in multivariate analysis.

Considered that switching to HD, kidney transplantation and lost to follow-up events had competing risks, Fine and Gray’s hazard model was also used for the competing risk analyses. After controlling competing risk events above, baseline LVH was still significantly risk factor for all-cause death (SHR, 2.645; 95% CI, 1.156–6.056; *p =* 0.021; SHR, 2.526; 95% CI, 1.097–5.817; *p =* 0.029 and SHR, 2.625; 95% CI, 1.144–6.027; *p =* 0.023, respectively). All the details were shown in Table 3.

**Table 3.** Predictors of overall and CVD mortality in 151 PD patients based on the results of Cox regression analysis and competing risk models.

	Univariate	multivariate							
		Model 0^a^		Model 1^b^		Model 2^c^		Model 3^d^	
	*p* value	AHR (95%CI)	*p* value	SHR (95%CI)	*p* value	SHR (95%CI)	*p* value	SHR (95%CI)	*p* value
**All-cause mortality**									
LA	Ref	Ref	–	Ref		Ref	–	Ref	–
L	0.983								
HA	0.500								
H	0.015								
Weekly renal Kt/V	0.103								
Hemoglobin (mg/L)	0.036	0.971 (0.946-0.997)	0.026	0.976 (0.946-1.007)	0.131	0.975 (0.947-1.005)	0.108	0.974 (0.945-1.003)	0.080
Triglyceride (mmol/L)	0.091								
Phosphate (mmol/L)	0.154								
LVH	0.028	3.716 (1.432-9.640)	0.007	2.645 (1.156-6.056)	0.021	2.526 (1.097-5.817)	0.029	2.625 (1.144-6.027)	0.023
LAE	0.023								
**CVD mortality**									
LA	Ref	Ref	–	Ref		Ref		Ref	
L	0.986								
HA	0.817								
H	0.014								
Weekly renal Kt/V	0.076								
Hemoglobin (mg/L)	0.093	0.962 (0.934-0.992)	0.013	0.979 (0.945-1.014)	0.226	0.978 (0.946-1.011)	0.190	0.976 (0.944-1.009)	0.148
Uric acid (μmol/L)	0.158								
Triglyceride (mmol/L)	0.162								
Cholesterol (mmol/L)	0.179								
Phosphate (mmol/L)	0.157								
LVH	0.076	3.923 (1.224-12.567)	0.021	0.422 (0.163-1.092)	0.076	2.346 (0.900-6.113)	0.081	2.442 (0.940-6.348)	0.067
LAE	0.441								

Bold indicates significance at *p* < 0.05.

^a^multivariable Cox proportional hazards regression model; ^b^competing risk model for switching to hemodialysis；^c^competing risk model for kidney transplant；^d^competing risk model for losing to follow up.

Abbreviations: AHR: adjusted hazard ratio; SHR: subdistribution hazard ratio; 95% CI: 95% confidence interval; CVD: cardiovascular disease; H: high; HA: high average; L: low; LA: low average; LAE: left atrial enlargement; LVH: left ventricular hypertrophy.

### Risk factor of LVH

**Figure 3.**
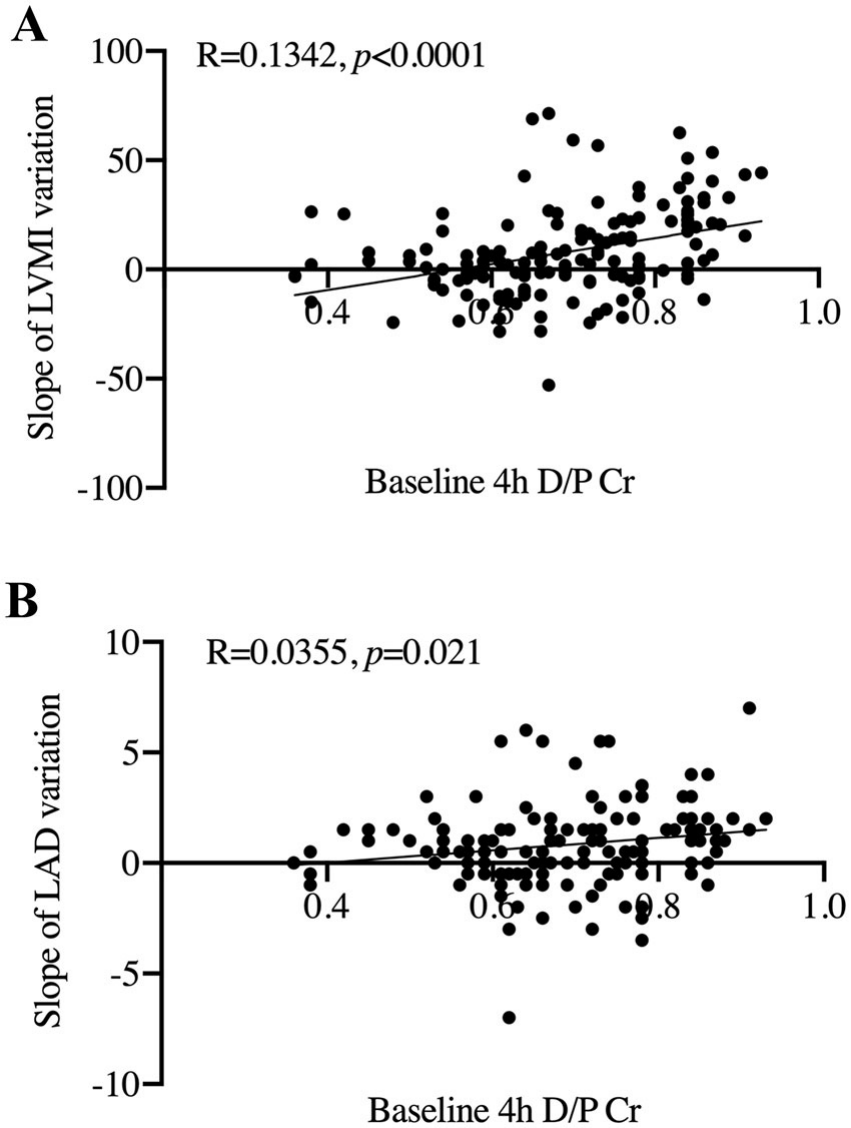
Scatter plot for correlation between baseline data and the rise slope of LVMI and LAD. (A) Baseline 4 h D/P Cr and the rise slope of LVMI. (B) Baseline 4 h D/P Cr and the rise slope of LAD. 4 h D/P Cr: dialysate/plasma creatinine at 4 h; LVMI: left ventricular mass index; LAD: left atrial diameter.

To explore the underlying risk factor of LVH and LAE, correlation analysis was conducted between baseline clinic data and the rise slope of LVMI as well as LAD. As shown in Figure 3, baseline 4 h D/P Cr levels showed a positive linear correlation with the rise slope of both LVMI (Figure 3(A)) and LAD (Figure 3(B)) (*p* < 0.0001 and *p =* 0.021, respectively).

Since this was a multicenter study, multivariate analysis was performed by using a generalized linear mixed model to evaluate the association between baseline clinic data and LVMI and LAD value 2 years after PD initiation. The results were showed in Table 4. baseline H and HA transport status was closely related to elevated LVMI (59.798 ± 12.15, *p <* 0.001 and 29.038 ± 8.76, *p =* 0.001, respectively) and LAD (11.316 ± 4.01, *p =* 0.005 and 6.424 ± 2.21, *p =* 0.004, respectively) 2 years after PD initiation. Apart from this, HbA1c levels showed negative association with LVMI (-7.994 ± 3.87, *p* = 0.042), Serum hs-CRP concentration showed positive associations with the value of LAD (0.125 ± 0.06, *p =* 0.038). Inverse correlation was also observed between LAD and non-diabetes (-3.532 ± 1.11, *p* = 0.002).

**Table 4.** Generalized linear mixed model (LVMI and LAD value as dependent variables after 2 years of PD initiation).

Fixed effects	LVMI		LAD	
	Coefficient (SE)	*p* value	Coefficient（SE）	*p* value
Age（years）	0.697 (4.02)	0.863	−0.065 (0.05)	0.199
Male	5.4707 (0.85)	0.488	1.817 (1.09)	0.072
L	24.447 (19.40)	0.211	−3.408 (3.83)	0.377
HA	29.038 (8.76)	**0.001**	6.424 (2.21)	**0.004**
H	59.798 (12.15)	**<0.001**	11.316 (4.01)	**0.005**
LA	Ref	–	Ref	–
Bp >140/90mmHg	−8.652 (11.37)	0.449	1.800 (1.53)	0.241
Non-Diabetes	5.704 (8.12)	0.484	−3.532 (1.11)	**0.002**
Weekly Peritoneal Kt/V	0.505 (3.59)	0.889	−0.359 (2.55)	0.886
Weekly renal Kt/V	4.287 (3.94)	0.279	2.449 (1.19)	0.053
UA (μmol/L)	0.012 (0.04)	0.767	−0.004 (0.01)	0.470
hs-CRP (mg/L)	5.064 (4.23)	0.234	0.125 (0.06)	**0.038**
Hb (mg/L)	−1.264 (3.73)	0.735	0.334 (0.03)	0.455
HbA1c (%)	−7.994 (3.87)	**0.042**	0.334 (0.45)	0.455
Alb (g/L)	0.433 (6.45)	0.947	−0.146 (0.17)	0.203
TG (mmol/L)	−0.862 (5.02)	0.864	−0.603 (0.71)	0.380
TC (mmol/L)	7.674 (4.45)	0.088	0.224 (0.56)	0.691
LDL (mmol/L)	−1.358 (4.11)	0.742	0.174 (0.55)	0.751
HDL (mmol/L)	−0.210 (4.22)	0.960	0.950 (1.78)	0.588
Serum P (mmol/L)	6.751 (3.63)	0.066	1.736 (1.00)	0.081
cSCa (mmol/L)	18.616 (16.24)	0.254	2.208 (2.24)	0.328
PTH (ng/L)	−4.098 (3.94)	0.301	0.001 (0.00)	0.734

Abbreviations: LVMI, left ventricular mass index; LAD, left atrial dimension; L, low; LA, low average; H, high; HA, high average; BP, blood pressure; Kt/V, urea clearance index; UA, uric acid; hs-CRP, high-sensitivity C-reactive protein; Hb, hemoglobin; HbA1c, glycosylated hemoglobin; Alb, albumin; TG, triglyceride; TC, total cholesterol; LDL, low density lipoprotein; HDL, high density lipoprotein; P, phosphate; cSCa, corrected serum calcium; PTH, parathyroid hormone.

Note: cSCa = serum Ca + 0.8_x_ (4.0-serum ALb) [if serum ALb < 4g/dL]. Bold indicates significance at *p < *0.05*.*

## Discussion

The current study highlighted the assessment of the prognoses of PD patients by the combination of baseline peritoneal transport characteristic with echocardiography parameters. We demonstrated that patients with LVH estimated by echocardiography at the initiation of PD may have worse prognosis.

As we known, LVH is common among patients with CKD or on dialysis. In a retrospective cohort study in Japan, concentric LVH at PD initiation was demonstrated as an independent risk factor for mortality (HR, 3.32; 95% CI, 1.13–9.70; *p* < 0.001) [18]. That conclusion was consistent with our result and the prevalence of baseline LVH in that cohort was even lower than ours (22% vs 36%). Some researchers suggested that LVH was the predominant characteristic of uremic cardiomyopathy which was often associated with diffuse myocardial fibrosis [19,20]. The uremic form of chronic cardiac fibrosis presents with uniform intramyocardial fibrosis, which is not the same as the endocardial to epicardial perivascular fibrosis seen in patients with hypertension and ischemic cardiomyopathies [21]. Histological features of endomyocardial biopsies in patients undergoing dialysis mainly included myocyte hypertrophy and diffuse fibrosis [22,23]. Diffuse myocardial fibrosis was associated with greater LV mass but not with risk factors for coronary atheroma, LV systolic dysfunction or angiographic coronary artery disease [20]. However, LV remodeling occur early during CKD. In a cross-sectional echocardiographic study of 3,487 patients, the detection rate of LVH could reach 32%, 48%, 57% and 75% for estimated GFR (eGFR) categories ≥60, 45 to 59, 30 to 44, and <30 mL/min/1.73 m^2^, respectively [9]. Therefore, we have reasons to believe that treatment should be focused on the earlier phases of CKD to prevent the progression of LVH.

LAE is closely related to LVH and diastolic dysfunction [24]. Several studies have demonstrated that left atrial volume index (LAVI) is an independent predictor of mortality in dialysis populations [25–28]. A prospective cohort study proved that changes in left atrial volume (LAV) had an independent association with cardiovascular outcomes (HR [1-mL/m^2.7^ per yr increase in LAV] 1.12; 95% CI, 1.05–1.20; *p* < 0.001), and the predictive ability was independent of left ventricular mass [29]. LA size as a predictor of adverse cardiovascular events may be associated with atrial fibrillation, acute myocardial infarction, heart failure, and cerebrovascular events [11]. In our study, LAE did not show a significant relationship with patients’ survival in the multivariate analysis, it still needed to be explored in larger samples.

The multivariate Cox regression model also showed the risk of all-cause and cardiovascular mortality decreasing with the rising hemoglobin. Considering the relatively high number of censored events competing for the primary outcome, no consistent conclusions have been drawn in competing risk models. However, numerous studies have identified anemia as an important risk factor for survival in dialysis patients [30,31]. Anemia is also proved to be a risk factor for LVH in both dialysis patients and non-dialysis CKD patients [32].

As in our results, baseline H transporters were found to exhibit an increased risk of overall and cardiovascular mortality in univariate analysis. Baseline H and HA transport status were closely associated with LVH and LAE in early stage of PD. Recently, Theerasak et al. retrospectively reviewed data on prevalent PD patients with repeat echocardiograms and extracellular water (ECW) measured by multifrequency bioelectrical impedance. They reported that ECW/height was independently associated with the percent change in LVMI (OR, 1.25; 95% CI, 1.08–1.36; *p =* 0.007), volume expansion was a more significant factor in determining LVH than blood pressure [33]. This result can also partly explain our findings. Fast transporters rapidly absorb dialysate glucose, leading to early eliminate of the osmotic gradient between dialysate and blood that is required to sustain ultrafiltration. Once the osmotic gradient is dissipated, the stimulus for ultrafiltration is gone, and ultrafiltration ceases. Therefore, patients with fast PSTR were more prone to develop into poor net ultrafiltration, low drain volume, and potential systemic volume expansion. Another disadvantage of fast PSTR was leading more and faster glucose absorption from higher concentrations of dialysate dextrose, which bring no benefits for patients to maintain good ventricular function. Hassan et al. reported that fluid overload, mean arterial pressure and mean LVMI were higher in patients treated with high peritoneal glucose dialysate group, whereas 24 h ultrafiltration, weekly Kt/V, serum albumin levels and RRF were better in low peritoneal glucose dialysate group [34]. Based on these findings, our results showed the value of baseline fast PSTR in predicting adverse outcomes due to its effects in promoting LVH and LAE. Actually, fast PSTR was also associated with many other adverse complications. Hu et al. recently reported that a higher baseline peritoneal transport status were risk factors for the first episode of peritonitis [35]. Therefore, peritoneal transport state may be a prognostic indicator as well as a therapeutic target for PD patients.

We also detected other clinical cardiovascular risk factors. High hs-CRP level was found to be a predictor of elevated LAD. Chronic inflammatory processes are common in ESKD. It is considered that CRP appears in every inflammatory state and is a sensitive biomarker of inflammation. In PD situation, many factors such as PD-associated peritonitis, long-term exposure to the bioincompatible solution, loss of RRF, fluid overload, and comorbid conditions like diabetes mellitus can increase chronic inflammation states. CRP has been widely proposed as a risk factor and an indicator of cardiovascular disease [36]. There is a strong association between the degree of CRP reduction and the corresponding reduction in CVD risk [37]. Chen et al. performed a retrospective cohort study included 574 dialysis patients (HD 347, PD 227) with median follow‐up of 3.5 years. They concluded that higher CRP quintiles were associated with increased risk of major adverse cardiovascular events in PD patients (*p* = 0.002) only, but not in hemodialysis patients [38]. Furthermore, higher HbA1c and non-diabetes patients were less associated with LVMI and LAD enlargement in our study. DM appeared to predict the risk of fatal cardiovascular events and all-cause mortality in PD patients [39]. But some dialysis patients tend to have false low HbA1c levels due to anemia and erythropoiesis-stimulating agents (ESAs) use. Actually, poor glycemic control and very low HbA1c levels attributed to poor nutritional status were associated with an increased mortality risk [40]. Due to the routine use of glucose-based fluids, glucose load is markedly higher in PD compared to HD patients. Novel non-glucose PD solutions have been reported to reduce cardiovascular events [41,42].

To the best of our knowledge, the studies focusing on the combination value of peritoneal transport characteristic and echocardiography for mortality risk are limited. This study particularly links baseline peritoneal transport characteristic and the changes in echocardiography together to provide a noninvasive assessment of all-cause and cardiovascular mortality risk in PD patients.

## Limitations

There are some limitations in this study. First, this was a retrospective study with a limited sample size. Some of the clinical data in the hospital recording system was incomplete. There were 33 (14.8%) patients be excluded due to lack of complete echocardiographic data. Therefore, the potential selection bias and confounding bias was inevitable. Secondly, although we further analyzed and compared slopes of echocardiography parameters changes in patients, only baseline PET results were enrolled in regression analysis, which did not cover the detail longitudinal D/P, Kt/V as well as RRF changes and their relations between echocardiography parameters variation during the observation period. In view of these confounding factors, additional larger multi-center studies are required to explore the correlation between peritoneal transfer characteristic and cardiac structure changes. Early intervention studies are urgently needed to improve the survival of PD patients.

## Conclusions

Baseline fast peritoneal solute transport rate may be an aggravating factor for left ventricular hypertrophy and predict poor outcomes for peritoneal dialysis patients. The findings offered that echocardiographic may be a useful adjunct for further prospective intervention trials in dialysis patients.

## Supplementary Material

Supplemental Material
