# Supplementary material for: A retrospective study of baseline peritoneal transport character and left ventricular hypertrophy in incident peritoneal dialysis patients: interrelationship and prognostic impacts
Source: Ren Fail. 2023 Jan 16;44(1):2073–84. doi: 10.1080/0886022X.2022.2148536 (PMC9848238; doi:10.1080/0886022X.2022.2148536)
Supplement: Supplemental Material [file IRNF_A_2148536_SM9398.pdf]

Supplementary table 1. The details on overall and CVD mortality in 151 PD patients

	L	LA	HA	H	Total
**All cause death**	0	4	7	10	21
peritonitis	0	1	1	0	2
other infections	0	0	1	1	2
stroke	0	0	1	0	1
**Cardiovascular death**	0	3	4	9	16
Heart failure	0	3	4	8	14
acute myocardial infarction	0	0	0	2	2

Abbreviations: CVD, Cardiovascular disease; H, high; HA, high average; L, low; LA, low average; PD, peritoneal dialysis.
